# Management of febrile neutropenia: consensus of the Brazilian Association of Hematology, Blood Transfusion and Cell Therapy - ABHH

**DOI:** 10.1016/j.htct.2024.11.119

**Published:** 2024-12-18

**Authors:** Marcio Nucci, Celso Arrais-Rodrigues, Maria Daniela Bergamasco, Marcia Garnica, Ana Beatriz Firmato Gloria, Mariana Guarana, Clarisse Machado, Jessica Ramos, Marco Aurelio Salvino, Belinda Simões

**Affiliations:** a Department of Internal Medicine, Hospital Universitário, Universidade Federal do Rio de Janeiro (UFRJ), Rio de Janeiro, Brazil; b Grupo Oncoclinicas, Rio de Janeiro, Brazil; c Department of Clinical and Experimental Oncology, Universidade Federal de São Paulo (Unifesp), São Paulo, Brazil; d Hospital Nove de Julho, Rede DASA, São Paulo, Brazil; e Hospital Israelita Albert Einstein, São Paulo, Brazil; f Complexo Hospitalar de Niterói – CHN-DASA, Niterói, Brazil; g Divisão de Hematologia da Federal da Universidade Federal de Minas Gerais (UFMG), Belo Horizonte, Brazil; h Grupo Oncoclínicas, Belo Horizonte, Brazil; i Hospital Universitário Gaffrée e Guinle, Universidade Federal do Estado do Rio de Janeiro (HUGG UFRJ), Rio de Janeiro, Brazil; j Instituto de Medicina Tropical da Universidade de São Paulo (IMT USP), São Paulo, Brazil; k Programa de Transplante de Células Hematopoiéticas do Hospital Amaral Carvalho, Jaú, Brazil; l Hospital Sírio-Libanês, São Paulo, Brazil; m PPGMS Universidade Federal da Bahia, Salvador, Bahia, Brazil; n Department of Medical Images, Hematology and Clinical Oncology, Ribeirão Preto Medical School – University of São Paulo, Brazil

**Keywords:** Neutropenia, Febrile neutropenia, Consensus, Management, Treatment

## Abstract

Febrile neutropenia is a major complication of the treatment of patients with hematologic diseases. Recent epidemiologic changes, with an increase in infection caused by drug-resistant bacteria, represent a major challenge for the proper management of febrile neutropenia. The impact of these changes in the epidemiology of infection may vary according to the region. In this document we present recommendations from the Infectious Diseases Committee of the Brazilian Association of Hematology, Blood Transfusion and Cell Therapy (ABHH) for the management of febrile neutropenia in hematologic patients. The consensus was developed by ten experts in the field, using the Delphi methodology. In the document we provide recommendations for the initial workup, prophylaxis, empiric antibiotic and antifungal therapy, modifications in the empiric regimen and criteria for discontinuing antimicrobial therapy.

## Introduction

The treatment of hematologic malignancies has changed substantially in recent years, with the incorporation of targeted therapies, cell therapy and advances in hematopoietic stem cell transplantation. On the other hand, changes in the epidemiology of bacterial infections, with the emergence of drug-resistant Gram-negative pathogens, has brought new challenges in the management of infection in patients with hematologic malignancies, including febrile neutropenia. The impact of these changes on the treatment of hematologic malignancies and on the epidemiology of infection may vary according to the region. Therefore, the simple incorporation of recommendations from guidelines created for one region may not apply to another region. In this document we present recommendations from the Infectious Diseases Committee of the Brazilian Association of Hematology, Blood Transfusion and Cell Therapy (ABHH) for the management of febrile neutropenia in hematologic patients.

## Methods

The group of panelists comprised all seven members of the Infectious Diseases Committee of the ABHH plus three non-members of the Committee who were invited to participate. The ten panelists comprised four infectious diseases specialists and six hematologists, all with expertise in the management of high-risk neutropenia, including patients with acute myeloid leukemia (AML) and acute lymphoid leukemia (ALL) receiving intensive chemotherapy and targeted therapies, and patients undergoing autologous and allogeneic hematopoietic stem cell transplantation (HSCT).

We used the Delphi methodology in the development of this consensus. The chairman (MN) prepared a series of statements regarding definitions for neutropenia and fever, antimicrobial prophylaxis, diagnostic procedures before fever, choice of the initial empiric antibiotic regimen, modifications in the initial regimen, monitoring during the episode of febrile neutropenia, use of non-prophylactic antifungal agents, and criteria for discontinuation of the antimicrobial agents. The panelists were asked to provide their opinions on each statement based on their experience and interpretation of the available literature.

For each statement, panelists were asked in a first round to provide responses to a multiple-choice questionnaire, rating their agreement on the question: strongly agree, agree, disagree, or strongly disagree. If at least 8 of the 10 panelists (80 %) agreed upon a statement it was considered to have achieved consensus, and did not go to a second round. In the second round, the same statements had two options: agree or disagree, and a comment field for the panelists to make comments regarding their agreement or disagreement, and to provide references supporting their comments. A third round was proceeded for questions not achieving consensus in Round 2. In the third round, a statement could be rephrased to accommodate disagreements in the first two rounds. Statements not achieving consensus in Round 3 were deemed non-agreement. The questions were developed in Google Forms and the answers were anonymous.

## Results

A total of 97 statements were created covering the following topics: general definitions (4 questions), antibacterial prophylaxis (5 questions), antifungal prophylaxis (20 questions), assessment of colonization by resistant bacteria (3 questions), procedures before starting empiric antibiotic therapy (7 questions), choice of the empiric antibiotic regimen (28 questions), procedures for persistent or recurrent fever (1 question), changes in the empiric antibiotic regimen (6 questions), use of biomarkers, images and invasive procedures (13 questions), use of non-prophylactic antifungal agents (3 questions), and discontinuation of antimicrobial therapy (7 questions). The consensus rate was 28 % after the first round, 56 % after the second round, and 67 % after the third round (Table 1). The lowest consensus rate was in the topic “choice of the empiric antibiotic regimen” (29 %).

**Table 1:** Overall rate of consensus per topic.

Topic	Number of questions	Consensus			
		First round	Second round	Third round	Total (%)
General definitions	4	1	3	0	4 (100)
Antibacterial prophylaxis	5	0	0	3	3 (60)
Antifungal prophylaxis	20	6	9	3	18 (90)
Assessment of colonization	3	1	1	0	2 (67)
Procedures before starting empiric antibiotic therapy	7	2	4	0	6 (86)
Choice of the empiric antibiotic regimen	28	3	0	5	8 (29)
Procedures for persistent or recurrent fever	1	1	0	0	1 (100)
Changes in the empiric antibiotic regimen	6	3	2	0	5 (83)
Use of biomarkers, images, and invasive procedures	13	4	5	0	9 (60)
Use of non-prophylactic antifungal agents	3	0	3	–	3 (100)
Discontinuation of antimicrobial agents	7	6	0	0	6 (50)
TOTAL	97	27	27	11	65 (67)

## General definitions

### Evidence summary

Patients with cancer who present with an absolute neutrophil count below 500/µL are at increased risk to develop severe infection.^1^ Regarding fever and persistent fever in neutropenic patients, the definitions are somehow arbitrary.

### Comments

The definition of neutropenia was considered as <500/µL with 90 % consensus, with the consideration that a patient with absolute neutrophil count above 500/µL but who had received chemotherapy recently with potential to induce neutropenia should be considered neutropenic for the purpose of triggering a protocol for febrile neutropenia (blood cultures, empiric antibiotic therapy and others). Fever defined as an axillary temperature >38 °C was accepted by 80 % of the panelists. Comments included a need of sustained fever (≥1 h) and the conversion of oral to axillary temperature (0.5 °C difference). Persistent fever was defined as fever that persists after three days of antibiotics with 100 % agreement. Recurrent fever was defined as a new episode of fever after 48 h afebrile with 80 % agreement.

### Recommendations

1. Neutropenia is defined as an absolute neutrophil count <500/µL.
2. Fever is defined as an axillary temperature >38 °C.
3. Persistent fever is defined as fever that persists after 3 days of antibiotic therapy.
4. Recurrent fever is defined as a new episode of fever after 48 h afebrile.

## Antibacterial prophylaxis

### Should neutropenic patients receive antibacterial prophylaxis?

#### Evidence summary

Several studies, including clinical trials and meta-analyses, have shown that fluoroquinolone prophylaxis reduces the incidence of febrile neutropenia and bloodstream infections in high-risk neutropenic patients (i.e., those with an expected duration of neutropenia >7 days).^2^, ^3^, ^4^, ^5^ In addition, a meta-analysis of randomized trials conducted until 2004 showed a reduction in mortality with antibiotic prophylaxis.^6^ More recently, the use of quinolones as prophylaxis in neutropenic patients has been questioned since some studies reported increased rates of resistance among Gram-negative organisms, especially quinolone-resistant bacteria.^7^^,^^8^ Moreover, in patients with baseline colonization by quinolone-resistant Gram-negative bacteria, the use of quinolones is not associated with better outcomes.^9^

#### Comments

The panel agreed (100 %) that quinolone prophylaxis should be considered in patients with AML receiving induction remission or after autologous HSCT, provided that the frequency of colonization and/or infection by resistant Gram-negative bacteria is not common. Regarding the use of quinolones during neutropenia occurring after consolidation therapy in AML, the panel did not reach consensus (70 % agreement), but considered giving prophylaxis if the patient is discharged after chemotherapy. Likewise, there was no consensus regarding the use of quinolones in the pre-engraftment phase of allogeneic HSCT. Comments included the lack of randomized trials in this scenario and concerns about drug interactions. The panel did not reach consensus (60 %) about which quinolone should be used (ciprofloxacin or levofloxacin).

#### Recommendations

1. The panel recommends against the routine/universal use of antibacterial prophylaxis.
2. The panel recommends the use of quinolone prophylaxis in centers with low incidence of colonization/infection by resistant Gram-negative bacteria.
3. The recommendation for quinolone prophylaxis is limited to patients with AML receiving intensive induction remission chemotherapy or after autologous HSCT.
4. The panel did not define a preference for a specific quinolone (levofloxacin or ciprofloxacin).
5. The panel recommends that if prophylaxis is given, close monitoring for the development of Gram-negative resistance should be carried out.

## Antifungal prophylaxis

### Should neutropenic patients receive antifungal prophylaxis?

#### Evidence summary

Patients with AML receiving intensive induction remission chemotherapy are at high risk to develop invasive fungal disease (IFD).^10^, ^11^, ^12^ In this setting, posaconazole is the drug of choice based on a randomized trial that evaluated primary antifungal prophylaxis in patients with AML or myelodysplasia syndrome (MDS) receiving intensive induction and showed a reduction of IFD and better overall survival compared to fluconazole or itraconazole.^13^ Moreover, large retrospective studies have also shown the benefit of posaconazole as antifungal prophylaxis in this population.^14^^,^^15^ Despite the lack of randomized trials in AML, voriconazole and more recently isavuconazole have been used as alternatives.^16^^,^^17^

Because of toxicity (especially hepatic) and drug interactions of the azoles, echinocandins have been used as prophylaxis in AML. A randomized study compared caspofungin with fluconazole in children and young adults with AML. Prophylaxis was given during neutropenia following each chemotherapy cycle. The incidence of IFD and invasive aspergillosis were significantly lower in the caspofungin arm, but the benefit was evident only after the third cycle of chemotherapy.^18^

The incidence of IFD in ALL varies according to the intensity of the chemotherapeutic regimen, with higher incidences in high-risk patients and in the setting of relapse.^19^^,^^20^ The use of azoles as prophylaxis in ALL is limited by their interference in the metabolism of vincristine. A randomized trial compared liposomal amphotericin B (5 mg/kg twice a week) with placebo. The incidence of IFD was slightly lower in the amphotericin B arm, but the difference was not statistically significant.^21^

Allogeneic HSCT recipients are at an increased risk to develop IFD, with two periods of high risk: the pre-engraftment period in the setting of severe neutropenia, and in the post-engraftment period, in the context of graft-versus-host disease (GvHD).^11^ In the pre-engraftment period, micafungin was compared with fluconazole in three randomized trials, none of which showed a significant reduction in the incidence of invasive aspergillosis in the micafungin arm.^22^, ^23^, ^24^ Voriconazole was evaluated as prophylaxis after allogeneic HSCT in two randomized trials. In the first, patients were randomized to receive itraconazole (intravenous/oral solution) or voriconazole from the pre-engraftment period until at least Day +100. The rates of IFD were low and similar in both arms.^25^ In the other study, patients received either fluconazole or voriconazole from the pre-engraftment period until at least Day +100. While the incidence of IFD was similar in both arms, the incidence of invasive aspergillosis was slightly lower in the voriconazole arm, but the difference was not statistically significant.^26^ Interestingly, in both arms patients were monitored with serum galactomannan performed twice weekly until Day +60, and once weekly thereafter until Day +100. A positive galactomannan triggered a search for invasive aspergillosis with chest computer tomography (CT) scans. Therefore, another interpretation of the trial was that fluconazole plus serial serum galactomannan was as good as voriconazole. A risk stratification approach has been advocated in AML to help clinicians to decide between these two strategies (primary prophylaxis with a mold-active antifungal agent versus prophylaxis with fluconazole plus serial monitoring with serum galactomannan), taking into consideration disease factors (probability of achieving complete remission with standard chemotherapy), host factors (performance status, comorbidities) and environmental exposure (care in rooms with or without high-efficiency particulate air filters).^27^

In autologous HSCT, fluconazole was compared with placebo in a randomized trial that also included patients with acute leukemia. The incidence of IFD (mostly candidemia) was lower in the fluconazole arm.^28^ In another study that also included allogeneic HSCT recipients, micafungin was compared with fluconazole, with similar rates of IFD.^25^

In ALL, although the incidence of IFD is relatively high,^12^ options for primary antifungal prophylaxis are limited because of drug interactions between azoles and vincristine, and because a randomized trial comparing liposomal amphotericin B with placebo did not show a reduction in the incidence of IFD in the amphotericin B arm,^29^

#### Comments

The panel agreed that in the induction remission of AML with intensive chemotherapeutic regimens, the choice for the prophylactic regimen should be based on a risk stratification strategy (100 %), and that posaconazole should be considered the agent of choice in patients considered at high risk for invasive aspergillosis (100 %). The panel agreed (80 %) that voriconazole is an option if posaconazole is indicated but not available. In patients who are not considered at a high risk for invasive aspergillosis, the panel agreed (80 %) that fluconazole is an option provided that serial serum galactomannan is available with results in a timely fashion (usually within 2–3 days). The panel agreed (100 %) that an echinocandin is an option if the patient develops liver toxicity or is receiving a targeted therapy to treat AML that interacts with the azole. The panel also commented that isavuconazole would be an option but acknowledged that there are just a few studies evaluating this agent as prophylaxis. The panel agreed that antifungal prophylaxis is usually not indicated in the consolidation phase of AML because the incidence of IFD is low.

The panel agreed (80 %) that in autologous HSCT, the decision for antifungal prophylaxis should be based on a risk stratification strategy, and if antifungal prophylaxis is considered, fluconazole is the agent of choice, considering that the incidence of invasive aspergillosis is usually low in this setting. For allogeneic HSCT, the panel also agreed (90 %) that the decision of giving an anti-mold agent or fluconazole should be based on a risk stratification strategy. The panel agreed (90 %) that voriconazol should be the anti-mold agent of choice, and that posaconazole is an option if voriconazole is not available. Moreover, the panel agreed (90 %) that an echinocandin is a suitable option if the patient develops liver toxicity. The panel commented that isavuconazole would be an option but acknowledged that there are just a few studies evaluating this agent as prophylaxis.

#### Recommendations

1. The panel recommends antifungal prophylaxis in the induction remission of AML.
2. The panel recommends a risk stratification strategy to define between an anti-mold agent or fluconazole.
3. The panel recommends that in patients receiving intensive chemotherapy for induction remission in AML/MDS, posaconazole is the anti-mold agent of choice.
4. The panel does not recommend routine antifungal prophylaxis in the consolidation phase of AML.
5. The panel recommends that if antifungal prophylaxis is considered after autologous HSCT, fluconazole is the agent of choice.
6. The panel recommends voriconazole as the anti-mold agent of choice after allogeneic HSCT.
7. The panel considers that an echinocandin is an option if the patient develops liver toxicity or is receiving a targeted therapy that interacts with the azole.

## Surveillance cultures

### Should surveillance cultures to assess colonization by resistant bacteria be performed in neutropenic patients?

#### Evidence summary

Surveillance cultures (usually nasal and anal swabs) are part of the strategies of infection control to prevent horizontal transmission of these organisms in the hospital. With the emergence of infections caused by multi-drug-resistant bacteria in neutropenic patients, the usefulness of surveillance cultures for resistant Gram-negative^30^, ^31^, ^32^, ^33^, ^34^ and Gram-positive^35^^,^^36^ bacteria have been evaluated. In general, these studies showed a high negative predictive value and variable positive predictive values. In other words, infection by a multi-drug-resistant organism is unlikely if the patient is not colonized.

#### Comments

The panel agreed (100 %) that a culture of an anal swab should be done on admission to evaluate if the patient is colonized by multi-drug-resistant Gram-negative bacteria. The panel also agreed (100 %) that weekly swabs should be obtained in centers with high incidence of colonization and/or infection by multi-drug-resistant Gram-negative bacteria. The panel did not reach a consensus (70 %) regarding assessment of colonization by methicillin-resistant *Staphylococcus aureus* (MRSA) with a nasal swab on admission.

#### Recommendations

1. The panel recommends a culture of an anal swab on admission to evaluate colonization by multi-drug-resistant Gram-negative organisms.
2. The panel recommends weekly cultures of anal swabs in centers with high incidence of colonization and/or infection by multi-drug-resistant Gram-negative organisms.

## Procedures before starting empiric antibiotic therapy

### What procedures should be performed before starting empiric antibiotic therapy in febrile neutropenic patients?

#### Evidence summary

The initial assessment of febrile neutropenic patient comprises history, physical examination, and blood cultures. It should start with a detailed medical history that includes co-morbidities, underlying disease and its status, the last chemotherapy regimen given and prior prophylactic antibiotics. Moreover, it is important to check prior episodes of infection and colonization to help guide therapy, especially if there is evidence of antibiotic-resistant organisms. Signs and symptoms should guide the physical examination looking especially for the most common sites of infection such as skin, and respiratory and gastrointestinal tracts.

Blood cultures are essential to detect a bloodstream infection. At least two sets of blood should be drawn, including peripheral vein and any available long-term catheter in the patient should be investigated as it can help to distinguish the source of bacteremia.^37^ Additional tests should be ordered if clinically indicated. In patients with respiratory symptoms, CT scans and a polymerase chain reaction (PCR) panel for respiratory viruses including COVID-19 is recommended. In contrast, the utility of chest X-ray is of little help as in neutropenic patients it can be normal even if there is a lung infection.^38^ Likewise, in the presence of a skin lesion, a biopsy is indicated urgently for microbiological documentation and due the risk of IFD.^39^

#### Comments

The panel agreed (80 %) that upon fever and before the start of empiric antibiotic therapy, two sets of blood cultures from peripheral vein and catheters should be obtained, and aerobic, anaerobic, and fungal bottles inoculated in an automated blood culture system. A few panelists commented that inoculation of fungal bottles could be considered in special situations but not routinely.

The panel agreed (80 %) that a chest X-ray should not be part of the routine procedures in febrile neutropenic patients due to its low sensitivity in detecting clinically relevant images. The panel also agreed (100 %) that a chest CT scan should be routinely obtained in febrile neutropenic patients who present signs or symptoms of respiratory disease. The panel did not reach a consensus regarding the performance of chest CT scan in asymptomatic febrile neutropenic patients or in patients with prior history of pneumonia.

The panel agreed (90 %) that an abdominal CT scan should be obtained in patients with abdominal symptoms or signs.

The panel agreed (90 %) that urine cultures should not be part of the routine workup in febrile neutropenic patients. Most panelists commented that in the absence of symptoms or some anatomic alteration in the urinary tract, a positive urine culture could merely represent asymptomatic bacteriuria.

#### Recommendations

1. The panel recommends the collection of two sets of blood cultures from a peripheral vein and a catheter to inoculate aerobic, anaerobic, and fungal bottles before the start of empiric antibiotic therapy in febrile neutropenic patients.
2. The panel recommends against the performance of a chest x-ray as part of the routine procedures in febrile neutropenic patients.
3. The panel recommends against urine cultures as part of the routine procedures in febrile neutropenic patients.
4. The panel recommends that a chest CT scan should be obtained in febrile neutropenic patients who present signs or symptoms of respiratory disease.
5. The panel recommends that an abdominal CT scan should be obtained in febrile neutropenic patients who present with abdominal signs or symptoms.

## Empiric antibiotic therapy

### What is the empiric antibiotic regimen for febrile neutropenic patients?

#### Evidence summary

The concept of empiric antibiotic therapy in febrile neutropenic patients was defined from the observations of a high early (within three days from the first fever) mortality rate in patients with bacteremia due to *Pseudomonas aeruginosa*, and a significant reduction in the death rate after the empiric initiation of an appropriate antibiotic regimen.^40^ Since then, various antibiotic regimens have been tested, always having as their backbone anti-*Pseudomonas* coverage. In the early development of anti-Gram-negative antibiotics, combinations of two (or even three) agents were given,^41^ but late in the 1990s, monotherapy became standard, usually cefepime, piperacillin-tazobactam or a carbapenem (imipenem, meropenem).^42^, ^43^, ^44^ These antibiotics have some differences in the clinical spectrum and side effects. For example, cefepime does not have anti-anaerobic activity while piperacillin-tazobactam and meropenem do. The use of anti-anaerobic antibiotics in neutropenic patients may reduce the diversity of the gut microbiome,^45^ resulting in various negative outcomes including GvHD and transplant-related mortality after allogeneic HSCT.^46^ Regarding toxicity, a randomized study in non-neutropenic patients comparing piperacillin-tazobactam with cefepime showed higher rates of neurologic complications (delirium and coma) in cefepime recipients.^47^ Finally, the overuse of meropenem may increase the risk of acquisition of a carbapenem-resistant Gram-negative infection.^48^

Patients with colonization by a resistant Gram-negative bacteria (such as extended-spectrum beta-lactamase [ESBL] or carbapenem-resistant enterobacteria [CRE]) are at an increased risk to develop bacteremia by the same colonizing agent.^49^^,^^50^ In these situations, there is a rationale for choosing an empiric antibiotic regimen that is active against the colonizing bacteria because the choice of an inappropriate anti-Gram-negative antibiotic regimen in febrile neutropenia is associated with higher mortality rates.^51^

#### Comments

The panel agreed (100 %) that cefepime and piperacillin-tazobactam are valid options as empiric antibiotic therapy in febrile neutropenic patients. The panel also agreed (90 %) that the choice between these two antibiotics should be based on local epidemiology and potential toxicity of each antibiotic. The panel agreed (90 %) that except in very special situations, meropenem should not be used as empiric antibiotic therapy in febrile neutropenic patients.

The panel did not agree regarding the choice of empiric antibiotic therapy in febrile neutropenic patients who are colonized by or had bacteremia by ESBL-producing enterobacteria in a previous episode of febrile neutropenia. Most of the panel (60 %) disagreed that patients colonized by this bacteria should receive a carbapenem upfront. Some of the panelists argued that most colonized patients do not have bacteremia caused by the same colonizing pathogen and that the empiric use of carbapenem may result in selective pressure and the emergence of infection by carbapenem-resistant bacteria. Likewise, the panel did not agree upon the choice of empiric regimen in patients who had bacteremia by ESBL-producing enterobacteria in a previous episode of neutropenia, with the same comments as in the case of colonization. However, most panelists considered the empiric use a carbapenem upfront in these circumstances only if the patient presented with hypotension or clinical deterioration.

The panel did not reach a consensus regarding the choice of the empiric antibiotic regimen in patients colonized by or who had a previous bacteremia by CRE, but in this situation, the majority (70 %) agreed that a special regimen should be chosen, considering the susceptibility profile of the colonizing organism. Likewise, the panel agreed (90 %) that if the patient received a special antibiotic regimen and blood cultures did not grow a resistant Gram-negative bacterium, the antibiotic regimen should be changed to cefepime or piperacillin-tazobactam.

#### Recommendations

1. The panel recommends cefepime or piperacillin-tazobactam as the empiric antibiotic therapy in febrile neutropenic patients; the choice between these two antibiotics should be based on local epidemiology and potential toxicity of each antibiotic.
2. The panel recommends against the routine use of a carbapenem as empiric antibiotic therapy in febrile neutropenic patients.
3. The panel recommends the use of a carbapenem as empiric antibiotic therapy in febrile neutropenic patients who present with hypotension or clinical deterioration and are colonized by ESBL-producing enterobacteria or had a bacteremia caused by this agent in a previous episode of febrile neutropenia.
4. The panel recommends the switch from a special antibiotic regimen to cefepime or piperacillin-tazobactam if blood cultures do not grow a resistant Gram-negative organism.

### Should vancomycin or another anti-methicillin-resistant S*taphylococcus aureus* antibiotics be part of the empiric antibiotic regimen in febrile neutropenic patients?

#### Evidence summary

The increase in the frequency of bacteremia caused by Gram-positive organisms in the late 1990s prompted investigators to test combinations of antibiotics with activity against these bacteria in the empiric antibiotic regimen of febrile neutropenic patients. However, randomized studies, summarized in a meta-analysis^52^ failed to show any significant benefit. On the other hand, guidelines published over a decade ago considered the use of vancomycin or other anti-MRSA antibiotics in certain circumstances such as suspected catheter-related infections, skin and soft tissue infections, pneumonia, or hemodynamic instability, but the level of evidence was weak.^53^ More recently, a series of epidemiologic studies have provided clinical data that challenge these recommendations. In the first study, factors associated with shock and early death (within four days of febrile neutropenia) were evaluated in a cohort of 1305 episodes of febrile neutropenia. By multivariate analysis pneumonia, shock and bacteremia caused by *P. aeruginosa* or *Klebsiella pneumoniae* were associated with early death, whereas bacteremia due to *Escherichia coli, Enterobacter* or *Acinetobacter* were predictive of shock. Importantly, infection by Gram-positive bacteria (including MRSA), catheter-related infections or skin or soft tissue infections were not associated with shock or early death, suggesting that lack of anti-MRSA coverage in the first fever does not result in an increased risk of early death.^54^ In another study, the impact of inappropriate empiric antibiotic therapy on mortality was evaluated in 1605 episodes of bloodstream infections in high-risk febrile neutropenic patients. Gram-positive bacteria accounted for 43 % of infections with the most frequent agents being coagulase-negative staphylococci, enterococci, and *S. aureus*. Inappropriate anti-Gram-positive coverage at first fever was not associated with higher mortality rates (16.5 % with inappropriate versus 14.4 % with appropriate antibiotic therapy).^51^ Another study evaluated the impact of the empiric antibiotic therapy on mortality of febrile neutropenic patients with bloodstream infections presenting with septic shock. The addition of an anti-Gram-positive antibiotic had no impact on mortality.^55^ Finally, a meta-analysis of trials comparing the treatment of febrile neutropenic patients with or without specific anti-Gram-positive coverage was conducted. The authors analyzed all patients as well as those with risk factors for Gram‐positive infections, including suspected catheter‐related infections, pneumonia, hypotension, and mucositis. The use of glycopeptides in the empiric regimen did not result in an improvement in the outcome.^56^ All these data indicate that empiric anti-MRSA coverage is not necessary in febrile neutropenic patients. This is especially true in patients who are not colonized by MRSA, as shown in a study of 194 patients with AML (484 admissions) who had a MRSA nasal swab during admission to treat febrile neutropenia. Among 471 admissions with negative nasal swabs, only three cases of infection were caused by MRSA (99 % negative predictive value).^35^

#### Comments

The panel agreed (100 %) that vancomycin (or another anti-MRSA antibiotic) should not be given routinely as part of the empiric antibiotic regimen. The panel also agreed (80 %) that vancomycin should not be given routinely to patients colonized by MRSA. The panel agreed that an anti-MRSA antibiotic should not be used empirically in the presence of pneumonia (80 %) or mucositis (80 %). By contrast, despite the bulk of epidemiologic studies showing that empiric vancomycin does not improve outcomes^51^^,^^54^, ^55^, ^56^ and the lack of evidence in support of its use, the panel did not reach a consensus regarding the empiric use of anti-MRSA antibiotics in the presence of suspected catheter-related infection (70 % voted against), skin or soft tissue infections (60 % voted against), or hypotension (70 % agreed).

The panel agreed (80 %) that vancomycin is the anti-MRSA antibiotic of choice in febrile neutropenic patients. The panel considered the use of other anti-MRSA antibiotics (teicoplanin, linezolid or daptomycin) only in special situations such as adverse drug events with vancomycin. The panel did not reach a consensus (70 %) regarding the need to monitor vancomycin serum levels. Those who disagreed commented that this measure should be only undertaken in case of a documented infection by MRSA and/or prolonged use of vancomycin.

#### Recommendations

1. The panel recommends against the routine empiric use of vancomycin (or other anti-MRSA antibiotics) in febrile neutropenic patients.
2. The panel recommends against the empiric use of vancomycin (or other anti-MRSA antibiotics) in febrile neutropenic patients who are colonized by MRSA.
3. The panel recommends against the empiric use of vancomycin (or other anti-MRSA antibiotics) in febrile neutropenic patients who have pneumonia or mucositis.
4. The panel recommends that once an anti-MRSA antibiotic is indicated, vancomycin is the agent of choice in febrile neutropenic patients.

## Monitoring patients during febrile neutropenia

### What biomarkers should I use during febrile neutropenia to monitor for bacterial infections?

#### Evidence summary

Various biomarkers of inflammation have been tested in febrile neutropenic patients, including C-reactive protein (CRP) and procalcitonin. Serial serum CRP predicted fever and bacteremia in neutropenic patients with acute leukemia in one study.^57^. In another study, high CRP serum levels were associated with prolonged duration of fever.^58^ A meta-analysis evaluated serum procalcitonin, CRP and interleukin-6 as predictors of bacteremia. Procalcitonin had the best positive likelihood ratio to confirm the diagnosis of bacterial infections.^59^

#### Comments

The panel did not reach a consensus (70 %) regarding the routine use of serial serum procalcitonin measurements in febrile neutropenic patients. Most panelists commented about its relatively high cost compared with CRP and that although it may help to indicate a bacterial infection or sepsis, its practical role has not been well established.

The panel agreed (90 %) that serial (three times weekly) serum CRP dosages may help to define strategies during febrile neutropenic. The panelists commented that decisions about changes in the empiric antibiotic regimen may be taken based on the kinetics of serum CRP (a rising curve suggesting a new infection and a flat curve suggesting no new infection).

#### Recommendations

1. The panel recommends obtaining serial serum CRP during an episode of febrile neutropenia.

### Should I obtain additional blood cultures in case of persistent or recurrent fever during neutropenia?

#### Evidence summary

Persistent fever or recurrent fever is frequent in febrile neutropenic patients especially if neutropenia is prolonged. In addition, the time to defervescence may vary, depending of the documentation of infection. Although there are many causes of fever, including mucositis, infusion of blood products, fever associated with the underlying disease, and drug reactions, breakthrough bacteremia is of concern because it may be associated with more resistant bacteria and higher mortality rates.^60^^,^^61^

#### Comments

The panel agreed (100 %) that in the case of persistent or recurrent fever (see definitions above), blood cultures should be obtained to diagnose breakthrough bacteremia. The panel agreed that the interval between blood cultures in patients who persist with fever should be defined on a case-by-case basis depending on the clinical situation.

#### Recommendations

1. The panel recommends obtaining blood cultures in neutropenic patients with persistent or recurrent fever during an episode of febrile neutropenia.

### When should I obtain images during febrile neutropenia?

#### Evidence summary

Pneumonia is a frequent infection in febrile neutropenic patients, and a chest CT scan is an important diagnostic tool.^62^ In high-risk neutropenic patients, the combination of chest CT scan and serial serum galactomannan represent the best strategy for the early diagnosis of invasive aspergillosis.^63^ In patients with clinical manifestations suggestive of typhlitis, an abdominal CT scan may show thickening of the bowel wall, confirming its diagnosis.^64^

#### Comments

The panel agreed (100 %) that a chest CT scan should be obtained in patients with persistent or recurrent fever even without respiratory symptoms. The panel also agreed (100 %) that a chest CT scan should be obtained in patients who present one or more positive serum galactomannan antigen tests.

The panel agreed (90 %) that during febrile neutropenia, an abdominal CT scan should be obtained if the patient presents with abdominal complains (pain, distention).

#### Recommendations

1. The panel recommends obtaining a chest CT scan in case of persistent or recurrent fever.
2. The panel recommends obtaining a chest CT scan in patients with one or more positive serum galactomannan antigen tests.
3. The panel recommends obtaining an abdominal CT scan if the patient presents with abdominal complains.

### When should I order invasive procedures during febrile neutropenia?

#### Evidence summary

In the investigation of lung infiltrates in febrile neutropenic patients, bronchoscopy with bronchoalveolar lavage represents a useful diagnostic tool when the etiology of lung infiltrates is uncertain.^65^ In patients with images suggestive of invasive aspergillosis, galactomannan in the bronchoalveolar lavage has an excellent accuracy in febrile neutropenic patients.^66^

Invasive fungal diseases, particularly invasive fusariosis, may present with metastatic skin nodules.^67^ An immediate biopsy with direct exam, culture and histopathology is mandatory under these circumstances, as a microscopic exam of the tissue may indicate the diagnosis within a few hours.^68^

#### Comments

The panel agreed (90 %) that bronchoalveolar lavage should be performed in febrile neutropenic patients who present with focal lung infiltrates suspicious of invasive aspergillosis and negative serum galactomannan. For patients with lung infiltrates and positive serum galactomannan, there was no consensus, with 60 % of panelists considering that bronchoalveolar lavage should not be performed because in this circumstance, the diagnosis of invasive aspergillosis would have already been established.

The panel agreed (90 %) that if the patient presents with skin lesions without a clear diagnosis, a skin biopsy should be performed immediately, especially in nodular lesions.

#### Recommendations

1. The panel recommends performing bronchoalveolar lavage in febrile neutropenic patients who present with focal lung infiltrates suspicious of invasive aspergillosis and negative serum galactomannan.
2. The panel recommends a skin biopsy in patients who appear with skin lesions (especially nodular lesions).

## Modifications in the empiric antibiotic regimen

### When should I add vancomycin (or another anti-MRSA antibiotic)?

#### Evidence summary

Two randomized placebo-controlled trials did not show any benefit of the empiric addition of a glycopeptide (vancomycin or teicoplanin) in persistently febrile neutropenic patients.^69^^,^^70^

#### Comments

The panel agreed (100 %) that empiric (negative blood cultures, no clinical signs of infection) vancomycin (or another anti-MRSA antibiotic) should not be given in cases of persistent or recurrent fever. The panel also considered inappropriate to add vancomycin empirically in patients colonized by MRSA with persistent or recurrent fever (90 % disagreement), but some panelists considered to add vancomycin empirically in patients who present clinical deterioration or if there is suspicion of catheter-related or skin or soft tissue infections, even without clinical data supporting these recommendations.

#### Recommendations

1. The panel recommends against the empiric use of vancomycin (or other anti-MRSA antibiotics) in patients with persistent or recurrent fever, even if the patient is colonized by MRSA.

### When should I change the beta-lactam antibiotic?

#### Evidence summary

There is no randomized study evaluating changing the beta-lactam antibiotic in persistently febrile neutropenic patients. In a prospective study, tigecycline was empirically added to the antibiotic regimen in persistently febrile neutropenic patients, resulting in defervescence in 68 % of patients.^71^ Therefore, no study has shown that the empiric change in the anti-Gram-negative coverage results in any benefit when persistent fever is the only manifestation. By contrast, although there are no randomized studies, it is common sense that a change in the beta-lactam regimen should be undertaken in the presence of new clinical signs of infection, clinical deterioration, or microbiologic documentation of infection.

#### Comments

The panel agreed (100 %) that in the case of worsening clinical conditions and/or the appearance of new clinical manifestations of infection, additional blood cultures and a change in the beta-lactam antibiotic (from cefepime or piperacillin-tazobactam to meropenem or another antibiotic regimen with a broader spectrum) should be undertaken even if the patient is afebrile.

Most panelists (70 %) disagreed regarding a change in the beta-lactam in cases of persistent or recurrent fever without new clinical manifestations of infection or clinical deterioration.

The panel agreed (80 %) that if the beta-lactam was changed empirically to a regimen with broader spectrum, a de-escalation to the original antibiotic regimen should be undertaken after 48–72 h if there is no documentation of infection caused by a Gram-negative organism exhibiting resistance to the first regimen (cefepime or piperacillin-tazobactam).

#### Recommendations

1. The panel recommends a change in the beta-lactam to a regimen of broader Gram-negative spectrum in case of clinical deterioration or the appearance of new clinical signs of infection, even if the patient is afebrile.

2. The panel recommends that if the beta-lactam is changed empirically to a regimen with broader spectrum, a de-escalation to the original antibiotic regimen should be undertaken after 48–72 h if there is no documentation of infection caused by a Gram-negative organism exhibiting resistance to the first regimen.

### When should I add anti-anaerobic coverage?

#### Evidence summary

Infections caused by anaerobe bacteria are uncommon in febrile neutropenic patients.^72^ However, in patients with typhlitis, an aerobe bacteria may be involved in the infection.^64^^,^^73^ A prospective cohort study evaluated the outcome of febrile neutropenic patients who presented with abdominal symptoms (abdominal pain, diarrhea, perianal pain) and received either cefepime plus metronidazole or piperacillin-tazobactam or meropenem monotherapy. The 28-day mortality rate was lower in patients receiving metronidazole.^74^

#### Comments

The panel agreed (90 %) that an anti-anaerobe antibiotic should be given in febrile neutropenic patients who present with clinical manifestations suspicious of typhlitis (abdominal distention, rebound tenderness) or with perianal pain.

### Recommendations

1. The panel recommends the start of an anti-anaerobe antibiotic in febrile neutropenic patients who present with clinical manifestations suspicious of typhlitis or with perianal pain.

## Fungal biomarkers and non-prophylactic antifungal use

### What fungal biomarkers should I use during febrile neutropenia?

#### Evidence summary

Serum galactomannan antigen testing is a backbone in the diagnosis of invasive aspergillosis.^75^ The use of serum galactomannan in neutropenic patients accelerates the time to diagnosis, with a potential to improve the outcome once invasive aspergillosis is diagnosed.^76^ In neutropenic patients not receiving anti-mold prophylaxis, serial (2–3 times weekly) galactomannan tests is the best strategy.^63^ However, the sensitivity of serum galactomannan reduces in patients receiving anti-mold azoles.^77^ In addition, the positive predictive value of the test may be lower, because the pre-test probability is lower. This has been reported with azoles^78^ and with echinocandins.^79^

Other fungal biomarker, 1,3-beta-d-glucan (BDG) can be used in febrile neutropenic patients. BDG is a component of the cell wall of various fungi, including *Candida* species, *Aspergillus* species, *Fusarium* species, *Pneumocystis jirovecii* and others. BDG is typically negative in cases of mucormycosis or cryptococcosis.^80^ The test is also positive in other conditions (false-positive) including the use of blood products, antibiotics, severe gastrointestinal mucositis, and bacteremia.^81^ A meta-analysis of cohort studies evaluating BDG as screening in patients with hematologic malignancies showed sensitivity and specificity of 70 % and 91 %, respectively.^82^

#### Comments

The panel agreed (90 %) that serial (2–3 times weekly) serum galactomannan antigen tests should be performed in neutropenic patients at high risk to develop invasive aspergillosis who are not receiving antifungal prophylaxis against *Aspergillus* species.

The panel agreed (90 %) that serial serum galactomannan should not be part of the routine in neutropenic patients receiving a mold-active azole such as voriconazole, posaconazole or isavuconazole because of the lower pre-test probability of aspergillosis in this scenario could result in false-positive results for serum galactomannan. In this situation, the panelists agreed (90 %) that serum galactomannan should be tested when invasive aspergillosis is suspected (e.g., recurrent, or persistent fever, respiratory symptoms such as a dry cough and pleuritic chest pain, or any suspicious image in chest CT scan), obtaining serum for three consecutive days. The panel did not reach a consensus (60 % agreement) about what strategy of galactomannan tests should be selected in patients receiving antifungal prophylaxis with an echinocandin.

The panel agreed (100 %) that the galactomannan antigen test should be performed in all bronchoalveolar lavage fluids obtained in the context of suspected invasive fungal pneumonia.

The panel did not reach a consensus (70 % agreement) about the role of obtaining serum levels of 1,3-beta-D glucan in febrile neutropenic patients at high risk to develop IFD. The main concerns were the sensitivity and low positive predictive value of the test, and the high cost.

#### Recommendations

1. The panel recommends serial (2–3 times weekly) serum galactomannan antigen tests for neutropenic patients at high risk to develop invasive aspergillosis who are not receiving antifungal prophylaxis against *Aspergillus* species.
2. The panel recommends against the serial serum galactomannan antigen test for neutropenic patients at high risk to develop invasive aspergillosis who are receiving an anti-*Aspergillus* antifungal agent.
3. The panel recommends the serum galactomannan antigen test (3 consecutive days) only upon suspicion of invasive aspergillosis in patients receiving an anti-*Aspergillus* antifungal agent.
4. The panel recommends galactomannan test in bronchoalveolar fluid obtained in the context of suspected invasive fungal pneumonia.

### When should I add a non-prophylactic antifungal agent?

#### Evidence summary

For many years, empiric antifungal therapy was considered standard of care in neutropenic patients who presented with persistent fever after 4–7 days of antibiotics.^83^^,^^84^ A problem with this strategy is that fever is too sensitive as a trigger to initiate a non-prophylactic antifungal agent, resulting in overuse of antifungals in febrile neutropenia. With the introduction of diagnostic tools for the diagnosis of IFD such as serum galactomannan, a preemptive strategy gained great interest. In this strategy, the trigger to initiate a non-prophylactic antifungal agent is the positivity of a biomarker, such as serum galactomannan. A large randomized study compared empiric versus preemptive therapy in high-risk febrile neutropenic adults receiving fluconazole prophylaxis. Preemptive therapy was not inferior to empiric therapy for the primary endpoint (6-wk survival), with a reduction in the use of non-prophylactic antifungal agents.^85^

#### Comments

The panel agreed (100 %) that patients with persistent or recurrent fever after 4–7 days of antibiotics should not routinely receive empiric antifungal therapy. The panelists agreed (100 %) that a preemptive strategy is more appropriate given the results of a large randomized trial comparing the two strategies and the high negative predictive value of the combination of serum galactomannan and chest CT scan. The panelists considered, however, that if results of serum galactomannan are not readily available and the patient is not in good clinical conditions, an antifungal agent may be started, and discontinued if a chest CT does not show suspicious images and serum galactomannan is negative at a later time.

The panelists agreed (90 %) that liposomal amphotericin B, caspofungin and voriconazole are valid options as empiric or preemptive antifungal therapy in febrile neutropenic patients. The panelists commented that the choice between these three antifungal agents should be based on which prophylactic regimen the patient was receiving, and the potential toxicity of each drug in each patient. Patients receiving a mold-active azole should receive liposomal amphotericin B. Patients not on antifungal prophylaxis or on fluconazole could receive either voriconazole or an echinocandin.

#### Recommendations

1. The panel recommends against the start of empiric antifungal therapy for neutropenic patients with persistent or recurrent fever after 4–7 days of antibiotic therapy.
2. The panel recommends a preemptive strategy of non-prophylactic antifungal therapy in neutropenic patients, using serum galactomannan and a chest CT scan.
3. The panel recommends liposomal amphotericin B, caspofungin or voriconazole as agents of preemptive antifungal therapy in febrile neutropenic patients.

## Discontinuation of antimicrobial therapy in febrile neutropenic patients

### When should I discontinue antibiotics in febrile neutropenia?

#### Evidence summary

Until recently, empiric antibiotic therapy was maintained at least until neutrophil recovery, even in afebrile patients with no documentation of infection. This recommendation was based on a study published in 1979 in which 33 neutropenic patients who were afebrile on Day 7 of antibiotics were randomized to discontinue (17 patients) or to keep (16 patients) the antibiotic regimen. A new fever occurred in seven of the 17 patients who discontinued versus none of the 16 who maintained the antibiotic until neutrophil recovery.^86^ More recently, a study randomized 157 neutropenic patients who were afebrile after 72 h of empiric antibiotic therapy, did not have any documentation of infection, and had normal vital signs, to continue (79 patients) or discontinue (78 patients) the empiric antibiotic regimen. Patients who discontinued the antibiotic regimen had less days on antibiotics, with no negative impact on the number of days with fever or mortality.^87^

#### Comments

The panelists agreed (90 %) that the antibiotic regimen should be discontinued when neutropenia is resolved (neutrophil count: >500/µL) and there is no documentation of infection (fever of unknown origin) even if the patient is still febrile. The panel also agreed (90 %) that upon neutrophil recovery, if there is documentation of infection during febrile neutropenia, the antibiotic regimen should be adjusted (usually de-escalation) and maintained until resolution of the documented infection.

The panelists agreed (90 %) that the empiric antibiotic regimen should be discontinued after four days in persistently neutropenic patients who do not have any documentation of infection and have normal vital signs (including heart and respiratory rates, blood pressure, oxygen saturation and diuresis). The panelists also agreed (80 %) that patients with persistent neutropenia and fever should not have the antibiotic regimen discontinued.

#### Recommendations

1. The panel recommends discontinuing the empiric antibiotic regimen if neutropenia resolves and there is no documentation of infection, even if the patient is still febrile.
2. The panel recommends that upon neutrophil recovery, if there is documentation of infection during febrile neutropenia, the antibiotic regimen should be adjusted and maintained until resolution of the documented infection.
3. The panel recommends discontinuing the empiric antibiotic regimen after four days in persistently neutropenic patients who do not have any documentation of infection and have normal vital signs.
4. The panel recommends continuing the empiric antibiotic regimen in patients who persist neutropenic and febrile.

### When should I discontinue antifungal agents in febrile neutropenia?

#### Evidence summary

In patients receiving non-prophylactic antifungal therapy when no IFD is documented, the antifungal agent is discontinued upon neutrophil recovery.^85^ For patients with documentation of IFD, the duration of antifungal therapy should be individualized, depending on the IFD diagnosed, response to treatment, the immune status of the patient and subsequent treatment for the underlying hematologic disease.^27^

#### Comments

The panelists agreed (100 %) that patients who received non-prophylactic antifungal therapy during neutropenia and do not have a diagnosis of IFD should have the antifungal discontinued upon neutrophil recovery, regardless of the duration of antifungal therapy. The panelists also agreed (100 %) that upon neutrophil recovery, if an IFD had been diagnosed, the duration of antifungal therapy should be determined on a case-by-case basis, considering the fungal disease diagnosed, immune status of the host and response to treatment.

#### Recommendations

1. The panel recommends discontinuing non-prophylactic antifungal therapy upon neutrophil recovery if there is no documentation of an IFD, regardless of the duration of antifungal therapy.

2. The panel recommends that upon neutrophil recovery, the duration of non-prophylactic antifungal therapy should be defined on a case-by-case basis, considering the fungal disease diagnosed, immune status of the host and response to treatment.

Table 2 shows all the recommendations for the management of febrile neutropenic patients suggested by the panelists in this consensus.

**Table 2:** Summary of recommendations for the management of febrile neutropenia.

Definitions
Neutropenia is defined as an absolute neutrophil count <500/µL
Fever is defined as an axillary temperature >38 °C
Persistent fever is defined as fever that persists after three days of empiric antibiotic therapy
Recurrent fever is defined as a new fever after 48 h afebrile
Antibacterial prophylaxis
The panel recommends against the routine/universal use of antibacterial prophylaxis
The panel recommends the use of quinolone prophylaxis in centers with low incidence of colonization/infection by resistant Gram-negative bacteria
The recommendation for quinolone prophylaxis is limited to patients with AML receiving intensive induction remission chemotherapy or after autologous HSCT
The panel did not define a preference for a specific quinolone (levofloxacin or ciprofloxacin)
The panel recommends that if prophylaxis is given, close monitoring for the development of Gram-negative resistance should be carried out
Antifungal prophylaxis
The panel recommends antifungal prophylaxis in the induction remission of AML
The panel recommends a risk stratification strategy to define between an anti-mold agent or fluconazole
The panel recommends that in patients receiving intensive chemotherapy for induction remission in AML/MDS, posaconazole is the anti-mold agent of choice
The panel does not recommend routine antifungal prophylaxis in the consolidation phase of AML
The panel recommends that if antifungal prophylaxis is considered after autologous HSCT, fluconazole is the agent of choice
The panel recommends voriconazole as the anti-mold agent of choice after allogeneic HSCT
The panel considers that an echinocandin is an option if the patient develops liver toxicity or is receiving a targeted therapy that interacts with the azole
Surveillance cultures
The panel recommends the culture of an anal swab on admission to evaluate colonization by multi-drug-resistant Gram-negative organisms
The panel recommends weekly cultures of anal swabs in centers with high incidence of colonization and/or infection by multi-drug-resistant Gram-negative organisms
Procedures before starting empiric antibiotic therapy
The panel recommends to collect two sets of blood cultures from a peripheral vein and catheters inoculate aerobic, anaerobic, and fungal bottles before the start of empiric antibiotic therapy in febrile neutropenic patients
The panel recommends against performing a chest x-ray as part of the routine procedures in febrile neutropenic patients
The panel recommends against urine cultures as part of the routine procedures in febrile neutropenic patients
The panel recommends that a chest CT scan should be obtained in febrile neutropenic patients who present signs or symptoms of respiratory disease
The panel recommends that an abdominal CT scan should be obtained in febrile neutropenic patients who present with abdominal symptoms or signs
Empiric antibiotic therapy
The panel recommends cefepime or piperacillin-tazobactam as empiric antibiotic therapy in febrile neutropenic patients; the choice between these two antibiotics should be based on local epidemiology and the potential toxicity of each antibiotic
The panel recommends against the routine use of a carbapenem as empiric antibiotic therapy in febrile neutropenic patients
The panel recommends the use of a carbapenem as empiric antibiotic therapy in febrile neutropenic patients who present with hypotension or clinical deterioration and are colonized by ESBL-producing enterobacteria or had a bacteremia caused by this agent in a previous episode of febrile neutropenia
The panel recommends the switch from a special antibiotic regimen to cefepime or piperacillin-tazobactam if blood cultures do not grow a resistant Gram-negative organism
Empiric anti-MRSA antibiotic
The panel recommends against the empiric use of vancomycin (or other anti-MRSA antibiotics) routinely in febrile neutropenic patients
The panel recommends against the empiric use of vancomycin (or other anti-MRSA antibiotics) in febrile neutropenic patients who are colonized by MRSA
The panel recommends against the empiric use of vancomycin (or other anti-MRSA antibiotics) in febrile neutropenic patients who have pneumonia or mucositis
The panel recommends that when an anti-MRSA antibiotic is indicated, vancomycin is the agent of choice in febrile neutropenic patients
Biomarkers for bacterial infections
The panel recommends obtaining serial serum CRP during an episode of febrile neutropenia
Additional blood cultures during febrile neutropenia
The panel recommends obtaining blood cultures in neutropenic patients with persistent or recurrent fever during an episode of febrile neutropenia
Images during febrile neutropenia
The panel recommends obtaining a chest CT scan in case of persistent or recurrent fever
The panel recommends obtaining a chest CT scan in patients with one or more positive serum galactomannan antigen tests
The panel recommends obtaining an abdominal CT scan if the patient presents with abdominal complains
Invasive procedures
The panel recommends performing bronchoalveolar lavage in febrile neutropenic patients who present with focal lung infiltrates suspicious of invasive aspergillosis and negative serum galactomannan test results
The panel recommends a skin biopsy in patients who appear with skin lesions (especially nodular lesions)
Empiric anti-MRSA during febrile neutropenia
The panel recommends against the empiric use of vancomycin (or other anti-MRSA antibiotics) in patients with persistent or recurrent fever, even if the patient is colonized by MRSA
Change of beta-lactam
The panel recommends a change in the beta-lactam to a regimen of broader Gram-negative spectrum antibiotic in case of clinical deterioration or the appearance of new clinical signs of infection, even if the patient is afebrile
The panel recommends that if the beta-lactam was changed empirically to a regimen with broader spectrum, a de-escalation to the original antibiotic regimen should be undertaken after 48–72 h if there is no documentation of infection caused by a Gram-negative organism exhibiting resistance to the first regimen
Anti-anaerobe
The panel recommends the start of an anti-anaerobe antibiotic in febrile neutropenic patients who present with clinical manifestations suspicious of typhlitis or with perianal pain
Fungal biomarkers
The panel recommends serial (2–3 times weekly) serum galactomannan antigen tests for neutropenic patients at high risk to develop invasive aspergillosis who are not receiving antifungal prophylaxis against *Aspergillus* species
The panel recommends against serial serum galactomannan antigen tests for neutropenic patients at high risk to develop invasive aspergillosis who are receiving an anti-*Aspergillus* antifungal agent
The panel recommends serum galactomannan antigen tests (3 consecutive days) only upon suspicion of invasive aspergillosis in patients receiving an anti-*Aspergillus* antifungal agent
The panel recommends a galactomannan test of bronchoalveolar fluid obtained in the context of suspected invasive fungal pneumonia
Non-prophylactic antifungal therapy
The panel recommends against the start of empiric antifungal therapy for neutropenic patients with persistent or recurrent fever after 4–7 days of antibiotic therapy
The panel recommends a preemptive strategy of non-prophylactic antifungal therapy in neutropenic patients, after a serum galactomannan test and a chest CT scan
The panel recommends liposomal amphotericin B, caspofungin or voriconazole as agents of preemptive antifungal therapy in febrile neutropenic patients
Discontinuation of antibiotic therapy
The panel recommends discontinuing the empiric antibiotic regimen if neutropenia resolves and there is no documentation of infection, even if the patient is still febrile
The panel recommends that upon neutrophil recovery, if there is documentation of an infection during febrile neutropenia, the antibiotic regimen should be adjusted and maintained until resolution of the documented infection
The panel recommends discontinuing the empiric antibiotic regimen after four days in persistently neutropenic patients who do not have any documentation of infection and have normal vital signs
The panel recommends continuing the empiric antibiotic regimen in patients who remain neutropenic and febrile
Discontinuation of antifungal therapy
The panel recommends discontinuing non-prophylactic antifungal therapy upon neutrophil recovery if there is no documentation of an invasive fungal disease, regardless of the duration of antifungal therapy
The panel recommends that upon neutrophil recovery, the duration of non-prophylactic antifungal therapy should be defined on a case-by-case basis, considering the fungal disease diagnosed, immune status of the host and response to treatment

## Conclusions

The recommendations presented in this document apply to febrile neutropenia that occurs after treatment for hematologic malignancies; they should be followed in the context of the epidemiology of each center. The document contains recommendations for antibacterial and antifungal prophylaxis, workup before starting empiric antibiotic therapy and throughout the course of febrile neutropenia, and guidance for the choice of empiric antibiotic regimen, as well as modifications to the regimen and criteria for discontinuation of antibiotics and antifungal agents.

## Funding

This research did not receive any specific grant from funding agencies in the public, commercial, or not-for-profit sectors.

## Conflicts of interest

None.
